# Butyrate Ameliorates Intestinal Epithelial Barrier Injury Via Enhancing Foxp3+ Regulatory T-Cell Function in Severe Acute Pancreatitis Model

**DOI:** 10.5152/tjg.2022.21307

**Published:** 2022-08-01

**Authors:** Shen Xiao, Sun Jing, Sun Jiakui, Zou Lei, Liu Ying, Liu Han, Mu Xinwei, Li Weiqin

**Affiliations:** 1 Department of Critical Care Medicine, Nanjing First Hospital, Nanjing Medical University, Nanjing, People’s Republic of China; 2 Department of General Surgery, Surgical Intensive Care Unit (SICU), Jinling Hospital, Medical School of Nanjing University, Nanjing, People’s Republic of China; 3 Department of Gastroenterology, Wuxi People’s Hospital Affiliated to Nanjing Medical University, Wuxi, People’s Republic of China

**Keywords:** Butyrate, intestinal barrier function, severe acute pancreatitis, treg cell

## Abstract

**Background:**

This study aimed to examine the effect of sodium butyrate on severe acute pancreatitis-related gut barrier injury in a rat model and explore its mechanism.

**Methods:**

Male rats randomly fell into 3 groups, that is, the control, the severe acute pancreatitis group, and the severe acute pancreatitis + butyrate group. Rats in the control group received sham operation, while rats in the severe acute pancreatitis group and severe acute pancreatitis + butyrate group received severe acute pancreatitis induction by intraductal infusion of 4% sodium taurocholate. After that, rats in the severe acute pancreatitis + butyrate group were fed with sodium butyrate solution with free access. Intestinal barrier injury was measured based on the expression of tight junction proteins by reverse transcription polymerase chain reaction, Western blotting assay as well as immunohistochemical staining. The variation of Treg cells was measured by reverse transcription polymerase chain reaction, Western blotting assay, immunohistochemical staining, and flow cytometry analysis.

**Results:**

Compared to rats in the control, rats in the severe acute pancreatitis group showed significantly higher pathohistological scores (*P* < .001) in the intestine, as well as decreased expression of occludin and ZO-1. While, rats in the severe acute pancreatitis + butyrate group showed mitigated histologic lesions (*P* < .05) and increased expressions of occludin and ZO-1. In addition, rats in the severe acute pancreatitis group showed the obvious reduction in the expressions of Foxp3 and GPR109a and the decreased percentage of Treg cells in the intestine (*P* < .001) compared to rats in the control. However, rats in the severe acute pancreatitis + butyrate group showed markedly increased expressions of Foxp3 and GPR109a and the upregulated percentage of Treg cells (*P* < .01).

**Conclusion:**

Butyrate could significantly mitigate the intestinal injury induced by severe acute pancreatitis, probably by inducing the differentiation of Treg cells.

## Introduction

Acute pancreatitis (AP) refers to a common critical disease of the digestive system, and its incidence has risen years by years.^1,2^ Though in most cases, AP is mild and self-limiting, it could be severe and life-threatening in nearly 5%-10% of the patients.^3^ On the whole, severe acute pancreatitis (SAP) has been complicated with multiple organ dysfunctions and local complications.^4^

Clinical and animal studies have confirmed the vital effect of gut in the occurrence and development of early excessive inflammatory responses and late sepsis in SAP.^5^ However, as revealed from recent studies, intestinal injury and intestinal infection attributed to increased permeability cause intestinal microflora translocation, and they are accompanied by immune-active cells in the intestinal wall and adjacent lymph nodes, that is, gut-associated lymphoid tissue (GALT).^6,7^

The latest studies reported that immune cells could be critical to the pathogenesis of SAP, which might be directly involved in the progression of SAP and mediate the damage of pancreas and extracellular organs.^8^ The immune cells involved in SAP consist of neutrophils, monocytes/macrophages, dendritic cells (DCs), mast cells, T lymphocytes, etc. T lymphocytes, especially CD4 + T lymphocytes, were confirmed to significantly impact the pathophysiological process of SAP.^9^ Regulatory T cells (Treg cells), pertaining to CD4 + T lymphocytes, have been reported to be vital for maintaining intestinal environmental homeostasis and immune tolerance in colitis, Crohn’s disease, and other disease models.^10,11^ However, researches on Treg cells primarily concentrated on the autoimmune pancreatitis (AIP) and chronic pancreatitis (CP), while studies on AP were rare.

Butyrate, one of the short chain fatty acids (SCFA), was critical for protecting intestinal barrier function and modulating intestinal immune function in numerous inflammatory bowel diseases (e.g., ulcerative colitis and Crohn’s disease^12-14^). Recent animal studies revealed that sodium butyrate could mitigate disease severity of SAP and its relevant organ injuries.^15,16^ However, the effect of sodium butyrate on intestinal barrier dysfunction induced by SAP has been rarely studied.

Accordingly, this study aimed to examine the protective effect of sodium butyrate on SAP-related gut barrier injury in the rat model and also explore the effect of Treg cell in the mechanism.

## MATERIALS AND METHODS

### Animals

All the protocols and experiments of this study were approved by the Animal Ethics Committee. Twenty-four male Sprague–Dawley (SD) rats [weight 200 ± 20 g; age 49-63 days] were bred and maintained under a specific pathogen-free (SPF) condition. Rats were maintained at 20-23°C under a 12-h day–night cycle and fed with standard rodent chow with free access to water. All the animal studies were conducted under the recommendations of the Care and Use of Experimental Animal.

### Experimental Groups

Sprague–Dawley rats randomly fell into 3 groups, that is, the control, the SAP group, and the SAP + butyrate group, with 8 rats in the respective group. After 12 hours of fasting, SAP was induced by intraductal infusion of 4% sodium taurocholate (S0900000, Sigma, Missouri, USA) following the description in the previous study.^17^ The rats in the control underwent sham operation, while SAP induction was performed for those in SAP and SAP + butyrate groups. Sodium butyrate (B5887, Sigma) was placed into the drinking water at a concentration of 150 mM (SB solution).^18^ The rats in the SAP + butyrate group and the SAP group were fed with SB solution or drinking water, respectively, with free access after the SAP induction. The therapeutic effect of butyrate was assessed at 72 hours after the SAP induction.

### Rodent Sample Collection

All rats were sacrificed at 72 hours after the model induction by intraperitoneally injecting 10% chloral hydrate at a lethal dose. Subsequently, several samples were collected (e.g., pancreas, distal ileum, colon, and peripheral blood). Parts of the pancreas, ileum, and colon samples were employed to conduct the flow cytometry analysis. Parts of these samples were flash frozen and then stored at –80°. The rest tissue samples were fixed with 10% paraformaldehyde and embedded with paraffin. Furthermore, peripheral blood samples were stored at –80° after being centrifugated at 3000 rpm for 10 min.

### Histology

The pancreatic, ileal, and colonic samples embedded in paraffin were cut into 6 μm-thick sections. Subsequently, the tissue sections were deparaffinized, rehydrated, and stained with hematoxylin and eosin (H&E) to be histologically examined. Two independent pathologists blinded to the treatment process examined each of the tissue samples under a confocal microscope (Olympus, Tokyo, Japan) and obtained an inflammation score by complying with the Schmidt’s, Chiu’s, and Berg’s method for the pancreas, ileum, and colon, respectively.^19-21^ Moreover, the tissue sections were stained with periodic acid schiff (PAS) staining and counterstained with hematoxylin to assess the glycogen deposits in the intestinal mucosa.

### Immunofluorescence

Additional sections were incubated with primary antibodies against forkhead box P3 (Foxp3) (mouse immunoglobin G [IgG], CatLog: sc-166212, Santa Cruz, California, USA), ZO-1: Zona Occludens 1 (ZO-1) (rat IgG, CatLog: sc-33725, Santa Cruz), and occludin (mouse IgG, CatLog: sc-133256, Santa Cruz), respectively, in phosphate-buffered saline (PBS) with 1% fetal calf serum (FCS) overnight at 4°. Next, the sections were washed and intubated with goat-anti-mouse (goat IgG, Catlog: ab150113, Abcam, Cambridge, UK) or goat-anti-rat secondary antibodies (goat IgG, Catlog: ab150165, Abcam) for 60 min. Images were obtained under a confocal microscope (Olympus).

### Enzyme-Linked Immunosorbent Assay (ELISA)

The concentrations of serum lipase, tumor necrosis factor (TNF)-α and interleukin (IL)-6 were determined with commercial enzyme-linked immunosorbent assay (ELISA) kits (R&D system, Minnesota, USA) by complying with the manufacturer’s protocol. Concentrations of the determined cytokines were set in duplicate supernatants through the comparison with standard curves generated with the provided recombinant cytokine.

### Preparation of Lymphocytes

Ileal and colonic lamina propria (LP) lymphocytes were prepared following to the procedures described previously.^22^ In brief, fresh tissues of ileum and colon were treated with Hanks Balanced Salt Solutions (HBSS, Gibco-Invitrogen, California, USA) containing 1 mM dithiothreitol (DTT) and 20 mM ethylene diamine tetraacetic acid (EDTA) for 30 min at 37° to remove the epithelial cells. Subsequently, collagenase solution containing 0.04 mg/mL collagenase type II (Biosharp, Basingstoke, UK) and 100 mM CaCl_2_ in HBSS were used to dissociate the tissues for 30 min at 37° to produce single-cell suspensions. Next, the single-cell suspensions were filtered and then washed with HBSS. Lastly, LP cells were further purified and then separated with a discontinuous Percoll (Sigma) gradient.

### Flow Cytometry Analysis

The following monoclonal antibodies, that is, anti-rat cluster of differentiation 4 (CD4) antibody (mouse IgG, Catlog: 11-0010-82, eBioscience, Basingstoke, UK), anti-rat cluster of differentiation 25 (CD25) antibody (mouse IgG, Catlog: 12-0390-82, eBioscience), and anti-rat Foxp3 antibody (rat IgG, Catlog: 56-5773-82, eBioscience), were conjugated with fluorescein isothiocyanate (FITC) allophycocyanin (APC), and phycoerythrin (PE) purchased from eBioscience. Lamina propria cells were washed with HBSS, and the isolated cells were suspended in the respective tube with 100 μL HBSS. Subsequently, the cells were stained with monoclonal antibodies, that is, anti-rat CD4 (mouse IgG, Catlog: 11-0010-82, eBioscience), anti-rat CD25 (mouse IgG, Catlog: 12-0390-82, eBioscience), and anti-rat Foxp3 (rat IgG, Catlog: 56-5773-82, eBioscience). For the intracellular staining of Foxp3, the cells were intubated with Fc gamma receptor (FcγR)-blocking monoclonal antibody before the staining for surface antigens under the manufacturer’s instructions.

### Quantification of Epithelial Apoptosis by Performing TUNEL Assay

Apoptotic cells were detected by performing terminal-deoxynucleoitidyl transferase mediated nick end labeling (TUNEL) assay based on the in situ cell death detection kit (Roche, Basel, Switzerland). The ileal and colonic sections were permeabilized with 0.1% sodium citrate and 1% Triton X-100 and stained by complying with the manufacturer’s protocols. All the sections were counterstained with 0.5 μM 4’,6-diamidino-2-phenylindole (DAPI) for 10 min and then mounted in antifading Fluorescent Mounting Medium (DakoCytomation, Copenhagen, Denmark). The images were captured under a confocal microscope (Olympus). Apoptotic rate was determined by determining the number of TUNEL-positive cells per field.

### Quantitative Real-Time Polymerase Chain Reaction (PCR) and Western Blotting Assay

The expression of gene transcript in the tissue was analyzed by real-time PCR with a StepOne™ Real-Time PCR System (Life technologies, California, USA). Quantitative real-time PCR was performed by complying with the procedures described previously.^23^ *ZO-1, Occludin*, and *Foxp3* gene expressions were studied, and the relative expressions were normalized by the expression of the housekeeping gene *actin*. The sequences of the primers comprised *actin*: sense CGTTGACATCCGTAAAGACCTC, antisense TAGGAGCCAGGGCAGTAATCT; *ZO-1*: sense CACGATGCTCAGAGACGAAGG, antisense TTCTACATATGGAAGTTGGGGATC;* Occludin*: sense GCAAAGTGAATGGCAAGAGATC, antisense CGTGTAGTCGGTTTCATAGTGGTC; *Foxp3*: sense, GTTTGCTGTGCGGAGACACC, antisense, CACTCTCCACTCGCACAAAGC.

Western blotting assay of the isolated intestinal and colonic tissues was performed following the previous description.^24^ The intensities of the band were analyzed and then quantified with AlphaEaseFC software (AlphaView, Cell Biosciences Inc., Santa Clara, USA).

### Statistical Analysis

The Statistical Package for Social Sciences version 22.0 software (IBM Corp.; Armonk, NY, USA) was employed to conduct statistical analyses. Data were expressed as means with standard errors (SEM). Statistical significance in groups was assessed with one-way ANOVA test. *P* value of <.05 was considered with statistical significance.

## Results

### Pathohistological Variations in the Pancreatic Tissues

**Supplementary Figure 1.**
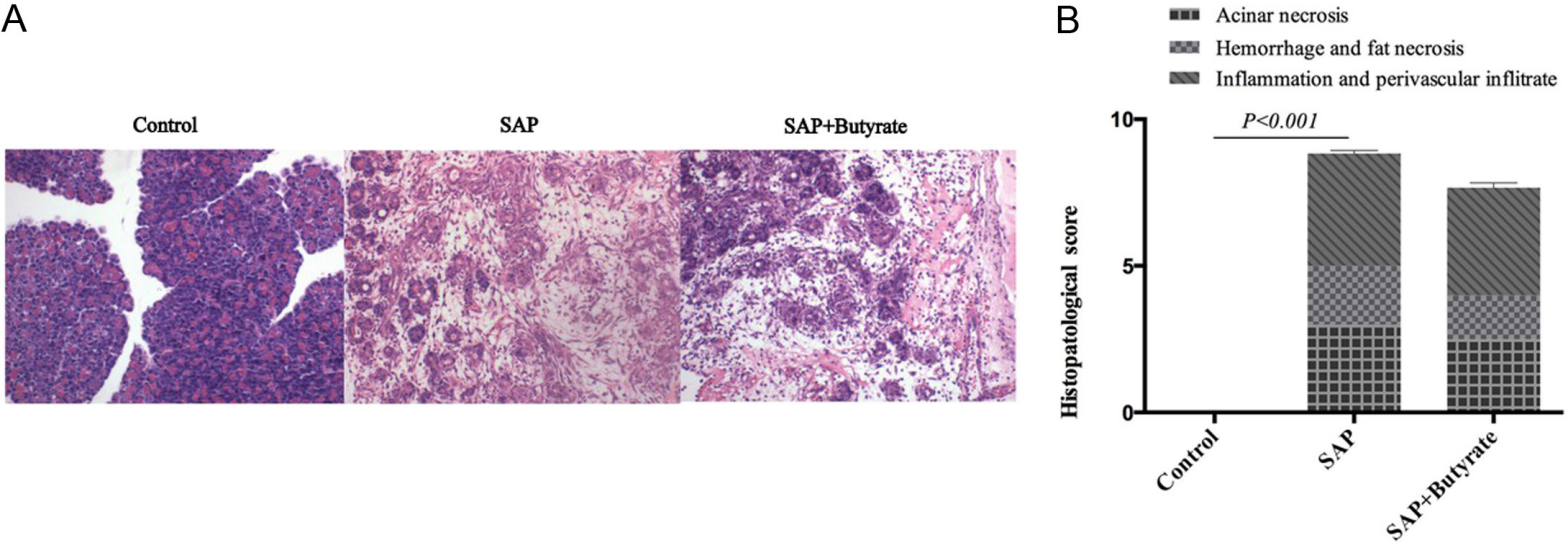
The effect of butyrate on the pancreas. Control: Control group, SAP: SAP group, SAP + butyrate: SAP + butyrate group. (A) Butyrate improved pathological injury in the pancreas. Paraffin sections of pancreas tissues were stained with H&E. (B) Histopathologic socres of the pancreas in each group. Control group versus SAP group: *P* < .001, Control group versus SAP + butyrate group: *P* > .05, SAP group versus SAP + butyrate group: *P* > .05. n = 8 in each group. SAP, severe acute pancreatitis; H&E, Hematoxylin and eosin.

Hematoxylin and eosin staining results showed that, compared to rats in the control, the normal structure of pancreatic acinus and the morphology was completely destroyed in rats with the SAP group (Supplementary Figure 1A). Large amounts of pancreatic acinar cells necrosis and inflammatory cells infiltration were detected, with widespread myofibroblasts. As a result, the mean pancreatic histological score of rats in the SAP group significantly exceeded that of the control (*P* < .001, Supplementary Figure 1B). However, the pathological variations were slightly mitigated in rats in the the SAP + butyrate group, with reduced pancreatic acinar cells necrosis and inflammatory cells infiltration (*P* > .05).

### Butyrate Mitigated Histologic Lesion of the Ileum and Colon

**Figure 1.**
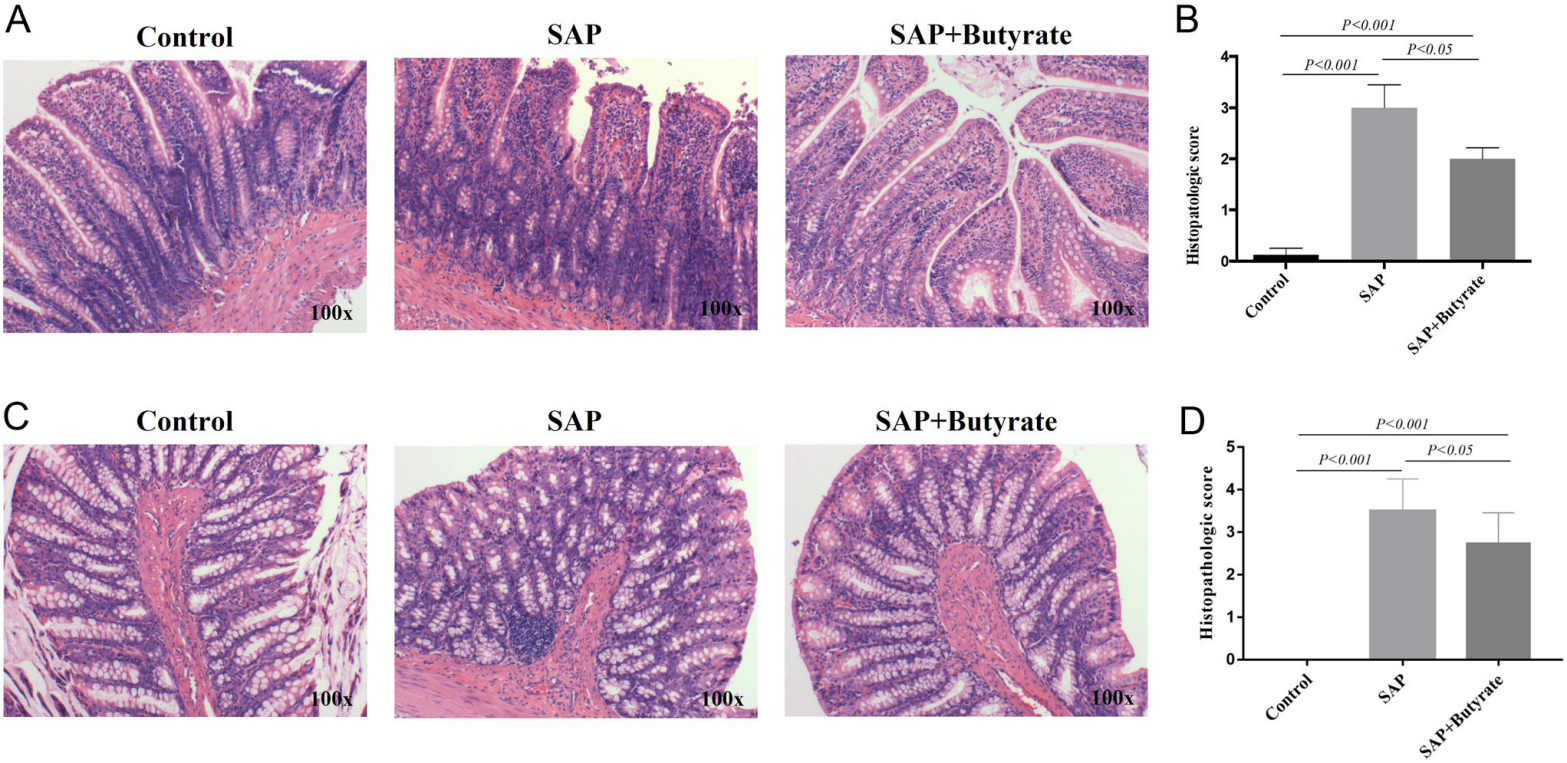
The effect of butyrate on intestinal injury. Control: Control group, SAP: SAP group, SAP + butyrate: SAP + butyrate group. (A) Butyrate markedly improved pathological inflammatory cell infiltration in the ileum. Paraffin sections of ileum tissues were stained with H&E. (B) Histopathologic socres of ileum in each group. Control group versus SAP group: *P* < .001, Control group versus SAP + butyrate group: *P* < .001, SAP group versus SAP + butyrate group: *P* < .05. (C) Butyrate markedly improved pathological inflammatory cell infiltration in the colon. Paraffin sections of colon tissues were stained with H&E. (D) Histopathologic socres of colon in each group. Control group versus SAP group: *P* < .001, Control group versus SAP + butyrate group: *P* < .001, SAP group versus SAP + butyrate group: *P* < .05. n = 8 in each group. SAP, severe acute pancreatitis; H&E, hematoxylin and eosin.

Next, the protective effects of sodium butyrate on the severity of SAP-related gut injury were assessed. Compared to the rats in the control, rats in the SAP group showed obviously more inflammatory cellular infiltration in the mucosa of both ileum and colon (Figure 1A and C) and markedly higher pathohistological scores (*P* < .001 for ileum and colon, Figure 1B and D). The histological lesions were more obvious in the ileum than those in the colon. However, rats in the SAP + butyrate group showed a significant decrease in ileac and colonic inflammation, less inflammatory cells infiltration, and markedly lower histological scores compared with those in the SAP group (*P* < .05 for ileum and colon, Figure 1).

### Butyrate Increased Epithelial Tight Junction Protein Expression and Maintained the Morphology in the Ileum and Colon of SAP Rats

**Figure 2.**
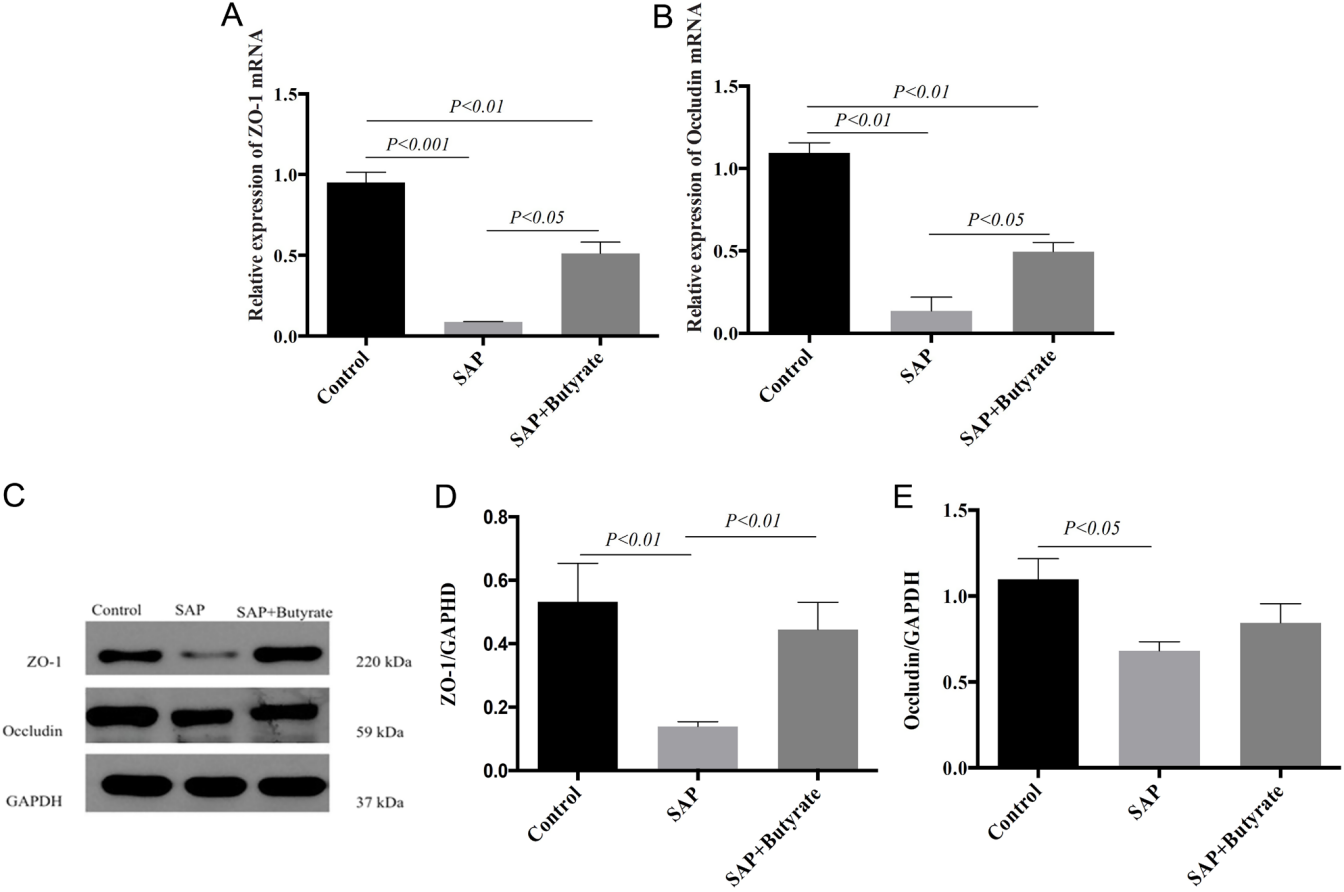
The effect of butyrate on the expression of tight junction proteins in the ileums. Control: Control group, SAP: SAP group, SAP + butyrate: SAP + butyrate group. (A) The mRNA expression of Zona Occludens 1 (ZO-1) detected by RT-PCR. Control group versus SAP group: *P* < .001, Control group versus SAP + butyrate group: *P* < .01, SAP group versus SAP + butyrate group: *P* < .05. (B) The mRNA expression of occludin detected by RT-PCR. Control group versus SAP group: *P* < .01, Control group versus SAP + butyrate group: *P* < .01, SAP group versus SAP + butyrate group: *P* < .05. (C) ZO-1 and occludin level by Western blotting. (D) ZO-1 protein relative to glyceraldehyde-3-phosphate dehydrogenase (GAPDH) level. Control group versus SAP group: *P* < .01, Control group versus SAP + butyrate group: *P* >.05, SAP group versus SAP + butyrate group: *P* < .01. (E) Occludin protein relative to GAPDH level. Control group versus SAP group: *P* < .05, Control group versus SAP + butyrate group: *P* > .05, SAP group versus SAP + butyrate group: *P* > .05. n = 8 in each group. SAP, severe acute pancreatitis; RT-PCR, reverse transcription polymerase chain reaction.

**Figure 3.**
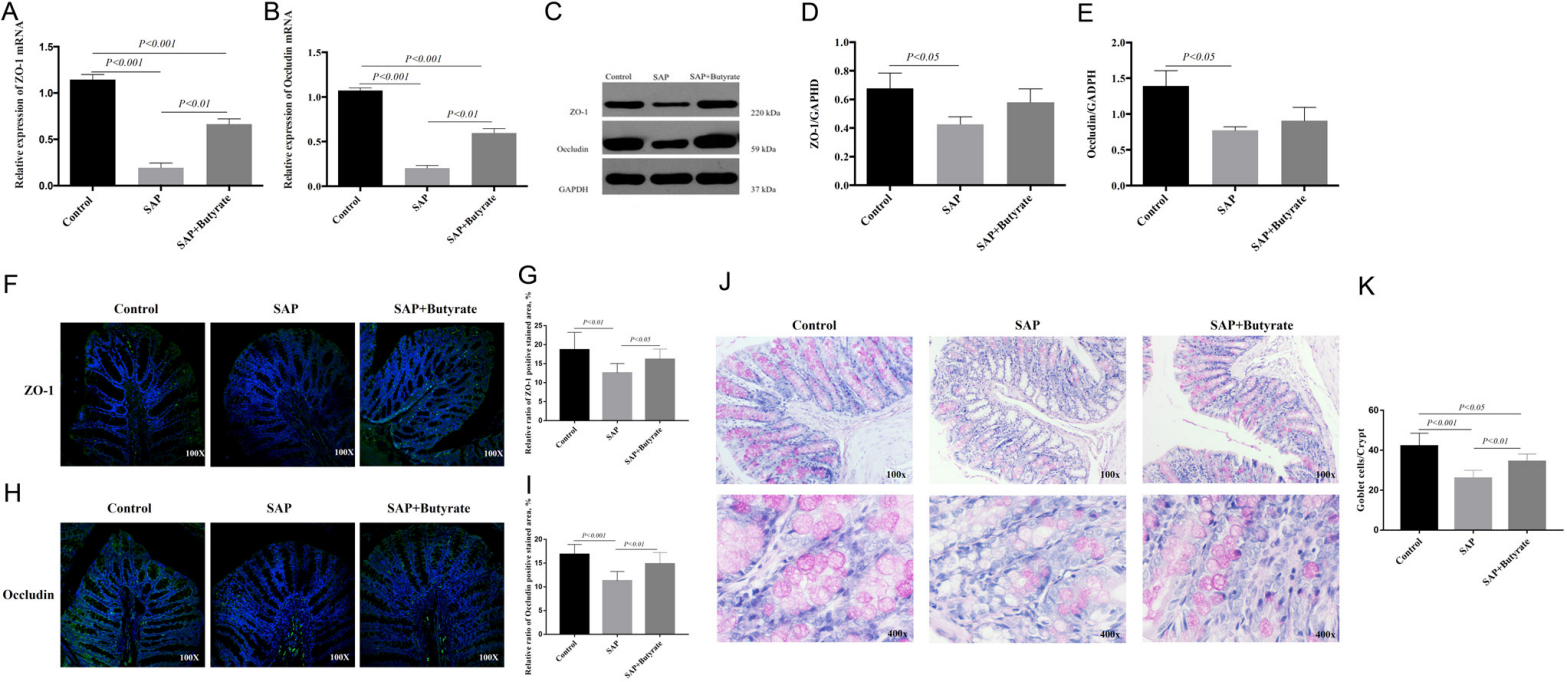
The effect of butyrate on the expression of tight junction proteins in the colons. Control: Control group, SAP: SAP group, SAP + butyrate: SAP + butyrate group. (A) The mRNA expression of ZO-1 detected by RT-PCR. Control group versus SAP group: *P* < .001, Control group versus SAP + butyrate group: *P* < .001, SAP group versus SAP + butyrate group: *P* < .01. (B) The mRNA expression of occludin detected by RT-PCR. Control group versus SAP group: *P* < .001, Control group versus SAP + butyrate group: *P* < .001, SAP group versus SAP + butyrate group: *P* < .01. (C) ZO-1 and occludin level by Western blotting. (D) ZO-1 protein relative to GAPDH level. Control group versus SAP group: *P* < .05, Control group versus SAP + butyrate group: *P* > .05, SAP group versus SAP + butyrate group: *P* > .05. (E) Occludin protein relative to GAPDH level. Control group versus SAP group: *P* < .05, Control group versus SAP + butyrate group: *P* > .05, SAP group versus SAP + butyrate group: *P* > .05. (F) The immunofluorescence detection of ZO-1 in the colon tissues. Green particles represented the positive change. (G) The quantitative analysis of immunofluorescence detection of ZO-1 in the colon tissues. The quantitative analysis of immunofluorescence was by ImageJ software. Control group versus SAP group: *P* < .01, Control group versus SAP + butyrate group: *P* > .05, SAP group versus SAP + butyrate group: *P* < .05. (H) The immunofluorescence detection of occludin in the colon tissues. Green particles represented the positive change. (I) The quantitative analysis of immunofluorescence detection of occludin in the colon tissues. The quantitative analysis of immunofluorescence was by ImageJ software. Control group versus SAP group: *P* < .001, Control group versus SAP + butyrate group: *P* > .05, SAP group versus SAP + butyrate group: *P* < .01. (J) PAS staining of the colon tissues. Red particles represented the positive change. (K) Quantification of the PAS-postive cells in each group. Control group versus SAP group: *P* < .001, Control group versus SAP + butyrate group: *P* < .05, SAP group versus SAP + butyrate group: *P* < .01. n = 8 in each group. SAP, severe acute pancreatitis; RT-PCR, reverse transcription polymerase chain reaction; PAS, periodic acid Schiff.

**Supplementary Figure 2.**
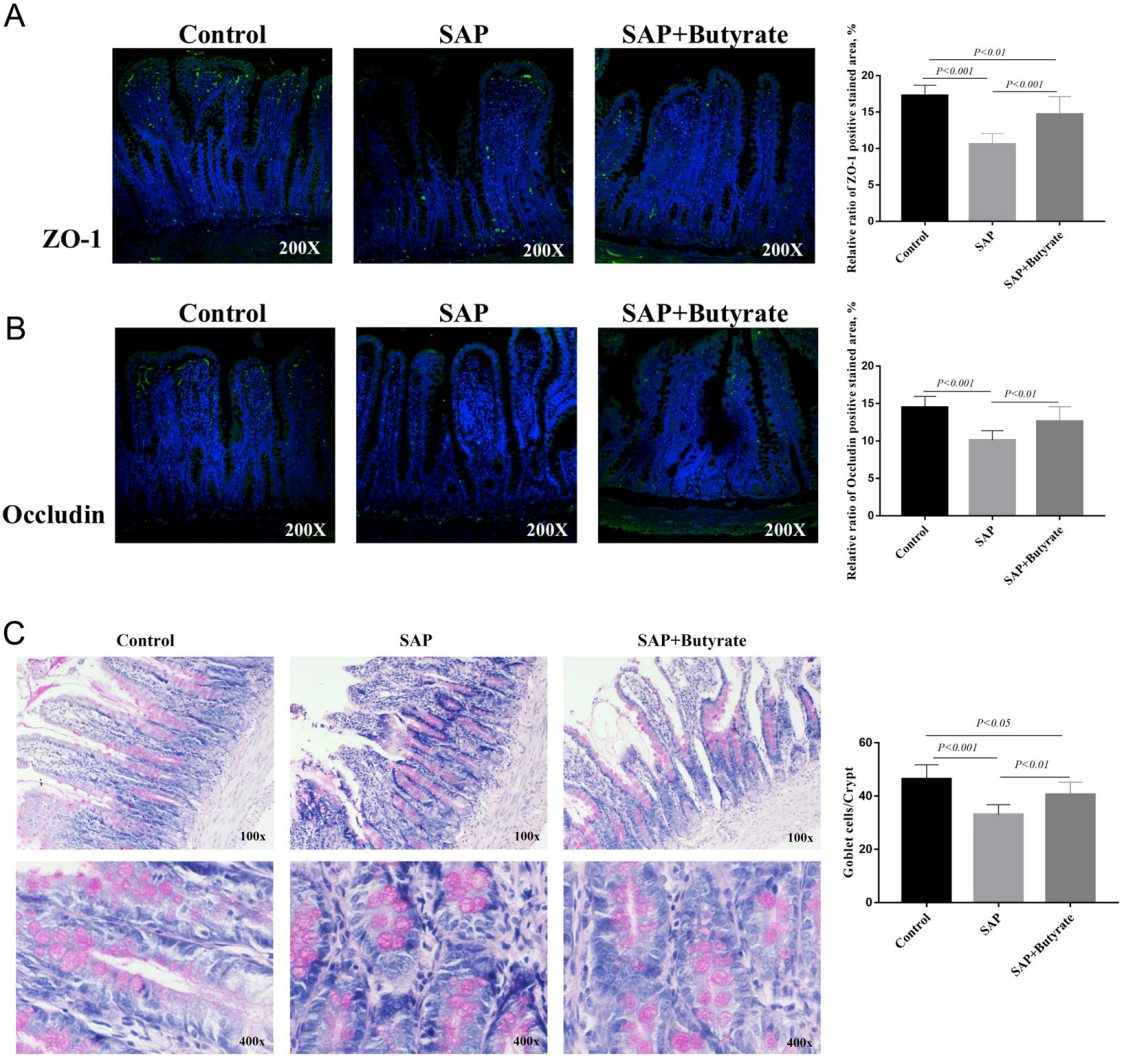
The effect of butyrate on the expression of tight junction proteins in the ileums. Control: Control group, SAP: SAP group, SAP + butyrate: SAP + butyrate group. (A) The immunofluorescence detection of ZO-1 in the ileum tissues. Green particles represented the positive change. The quantitative analysis of immunofluorescence was by ImageJ software. Control group versus SAP group: *P* < .001, Control group versus SAP + butyrate group: *P* < .01, SAP group versus SAP + butyrate group: *P* < .001. (B) The immunofluorescence detection of occludin in the ileum tissues. Green particles represented the positive change. The quantitative analysis of immunofluorescence was by ImageJ software. Control group versus SAP group: *P* < .001, Control group versus SAP + butyrate group: *P* > .05, SAP group versus SAP + butyrate group: *P* < .01. (C) PAS staining of the ileum tissues. Red particles represented the positive change. Control group versus SAP group: *P* < .001, Control group versus SAP + butyrate group: *P* < .05, SAP group versus SAP + butyrate group: *P* < .01. n = 8 in each group. SAP, severe acute pancreatitis.

Occludin and ZO-1 were assessed as 2 important proteins of intestinal mechanical barrier. The mRNA expressions of ZO-1 and occludin were significantly decreased in the ileum of the rats in the SAP group compared with those in the control (*P* < .001 and *P* < .01 for ZO-1 and occludin, respectively, Figure 2A and B), indicating impaired gut barrier function in SAP rats. The change in the colon was consistent with the ileum (*P* < .001 for both ZO-1 and occludin, Figure 3A and B). Western blotting assay also confirmed decreased expression of ZO-1 and occludin in the ileum of the rats in the SAP group than those in the control (*P* < 0.01 and *P* < 0.05 for ZO-1 and occludin, respectively, Figure 2C-E). Moreover, the identical variations were suggested in the colon (*P* < .05 for both ZO-1 and occludin, Figure 3C-E). However, rats in the SAP + butyrate group showed increased expressions of ZO-1 and occludin and ZO-1 at mRNA and protein levels compared to rats in the SAP group, though the differences were not statistically significant in protein level (Figure 2A-E and 3A-E). In addition, immunofluorescence staining also showed that the density of ZO-1 and occludin in the colon was markedly lower in the rats of SAP group than those in the control (*P* < .01 and *P* < .001 for ZO-1 and occludin, respectively, Figure 3F-I) but was improved significantly in the rats in the SAP + butyrate group (*P* < .05 and *P* < .01 for ZO-1 and occludin, respectively). The same variations were also shown in the ileum of the rats (Supplementary Figure 2A and B).

Goblet cells, as a key component of the intestinal mucosal immunity barrier, secreted mucins and large glycoproteins to protect the mucous membranes. Periodic acid schiff staining revealed a significant reduction of goblet cells in the colon and ileum of the rats in the SAP group compared to those in the control (*P* < .001 for both colon and ileum, Figure 3J-K and Supplementary Figure 2C), indicating impaired mucosal immunity barrier in SAP. However, the goblet cells were well restored in the rats of the SAP + butyrate group compared to those of the SAP group (*P* < .01 for both colon and ileum).

### Butyrate Increased Treg Cells in the Ileum and Colon of SAP Rats

**Figure 4.**
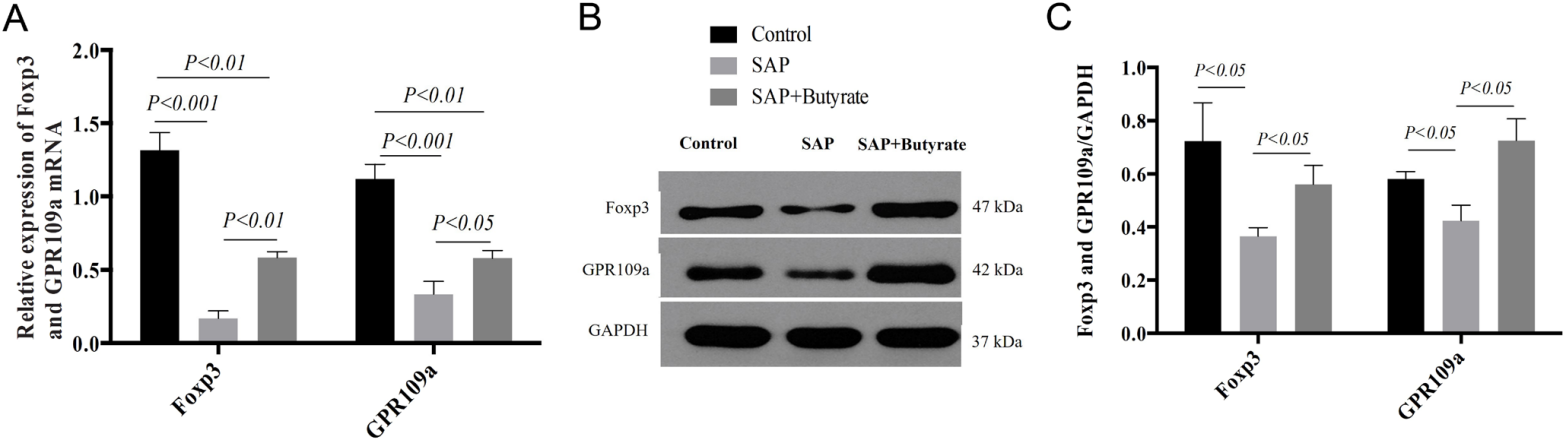
The effect of butyrate on the expression of Foxp3 and GPR109a in the ileums. Control: Control group, SAP: SAP group, SAP + butyrate: SAP + butyrate group. (A) The mRNA expression of Foxp3 and GPR109a detected by RT-PCR. Expression of Foxp3: Control group versus SAP group: *P* < .001, Control group versus SAP + butyrate group: *P* < .01, SAP group versus SAP + butyrate group: *P* < .01. Expression of GPR109a:Control group versus SAP group: *P* < .001, Control group versus SAP + butyrate group: *P* < .01, SAP group versus SAP + butyrate group: *P* < .05. (B) Foxp3 and GPR109a level by Western blotting. (C) Foxp3 and GPR109a protein relative to GAPDH level. Expression of Foxp3: Control group versus SAP group: *P* < .05, Control group versus SAP + butyrate group: *P* > .05, SAP group versus SAP + utyrate group: *P* < .05. Expression of GPR109a: Control group versus SAP group: *P* < .05, Control group versus SAP + butyrate group: *P* > .05, SAP group versus SAP + butyrate group: *P* < .05. n = 8 in each group. SAP, severe acute pancreatitis.

**Figure 5.**
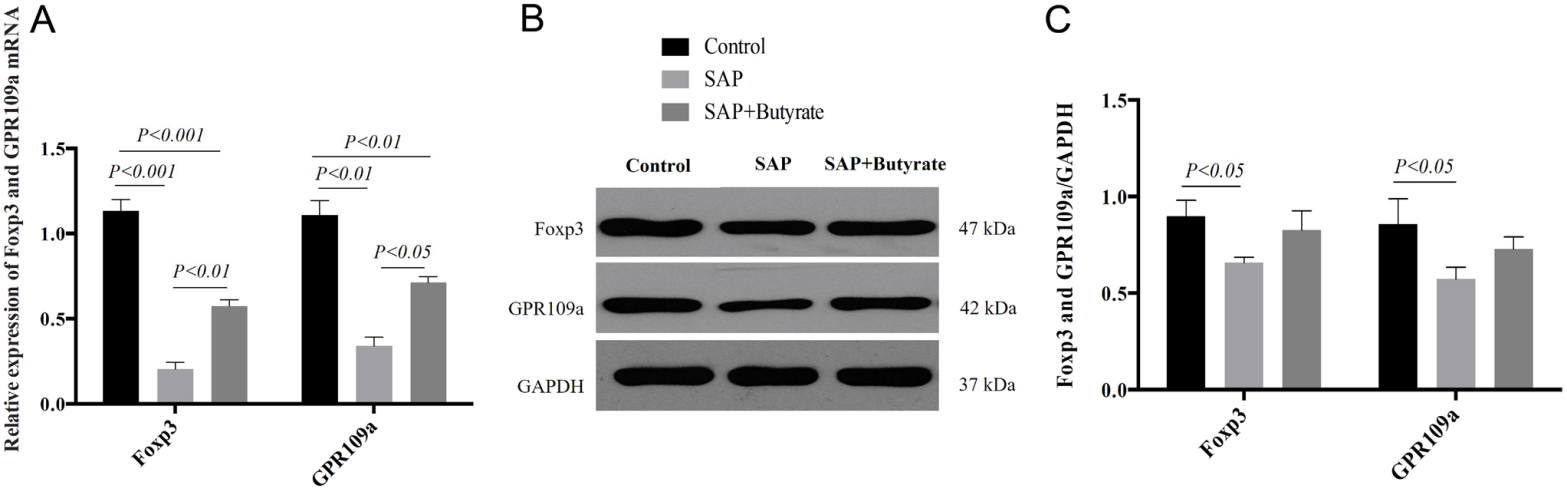
The effect of butyrate on the expression of Foxp3 and GPR109a in the colons. Control: Control group, SAP: SAP group, SAP + butyrate: SAP + butyrate group. (A) The mRNA expression of Foxp3 and GPR109a detected by RT-PCR. Expression of Foxp3: Control group versus SAP group: *P* < .001, Control group versus SAP + butyrate group: *P* < .001, SAP group versus SAP + butyrate group: *P* < .01. Expression of GPR109a: Control group versus SAP group: *P* < .01, Control group versus SAP + butyrate group: *P* < .01, SAP group versus SAP + butyrate group: *P* < .05. (B) Foxp3 and GPR109a level by Western blotting. (C) Foxp3 and GPR109a protein relative to GAPDH level. Expression of Foxp3: Control group versus SAP group: *P* < .05, Control group versus SAP + butyrate group: *P* > .05, SAP group versus SAP + butyrate group:* P* > .05. Expression of GPR109a: Control group versus SAP group: *P* < .05, Control group versus SAP + butyrate group: *P* > .05, SAP group versus SAP + butyrate group: *P* > .05. Control group versus SAP group: *P* < .001, Control group versus SAP + butyrate group: *P* < .05, SAP group versus SAP + butyrate group: *P* < .001. n = 8 in each group. SAP, severe acute pancreatitis; RT-PCR, reverse transcription polymerase chain reaction.

The expressions of Foxp3 (marker for Treg cells) and GPR109A (a receptor for butyrate in the colon) were assessed in the intestine of the rats. Reverse transcription polymerase chain reaction analysis revealed that the expressions of Foxp3 in ileum and colon of the rats in the SAP group were downregulated significantly (*P* < .001 for ileum and colon, Figures 4A and 5A). Western blotting assay confirmed the identical variation in protein level (*P* < .05 for ileum and colon, Figures 4B-C and 5B-C). Consistent with Foxp3, the expression of GPR109a was also markedly downregulated in the rats of the SAP group in the ileum (*P* < .001 at mRNA level, *P* < .05 at protein level, Figure 4A-C) and in the colon (*P* < .01 at mRNA level, *P* < .05 at protein level, Figure 5A-C). Whereas, compared to rats in the SAP group, the expression of Foxp3 was significantly increased at mRNA and protein levels in the ileum of the rats in the SAP + butyrate group (*P* < .01 at mRNA level, *P* < .05 at protein level, Figure 4A-C). The identical variation was also identified in the colon (*P* < .01 at mRNA level, *P* > .05 at protein level, Figure 5A-C), though the difference was not statistically significant at the protein level of the colon. Consistent with Foxp3, the expression of GPR109a was also markedly upregulated in the ileum (*P* < .05 at mRNA and protein levels, Figure 4A-C) and colon (*P* < .05 at mRNA level, *P* > .05 at protein level, Figure 5A-C) of the rats in the SAP + butyrate group compared with those in the SAP group.

**Supplementary Figure 3.**
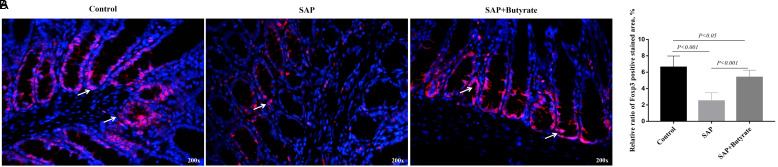
The effect of butyrate on the expression of Foxp3 in the colons. Control: Control group, SAP: SAP group, SAP + butyrate: SAP + butyrate group. (A) The immunofluorescence detection of Foxp3 in the colon tissues. Red particles (arrow) represented the positive change. (B) The quantitative analysis of immunofluorescence detection of Foxp3 in the colon tissues. The quantitative analysis of immunofluorescence was by ImageJ software. Control group versus SAP group: *P* < .001, Control group versus SAP + butyrate group: *P* < .05, SAP group versus SAP + butyrate group: *P* < .001. n = 8 in each group. SAP, severe acute pancreatitis.

Immunofluorescence staining indicated that the density of Foxp3 expression in the colon of the rats in the SAP group were significantly lower than those in the control (*P* < .001), which was improved noticeably in rats of the SAP + butyrate group (*P* < .001, Supplementary Figure 3A and B).

**Figure 6.**
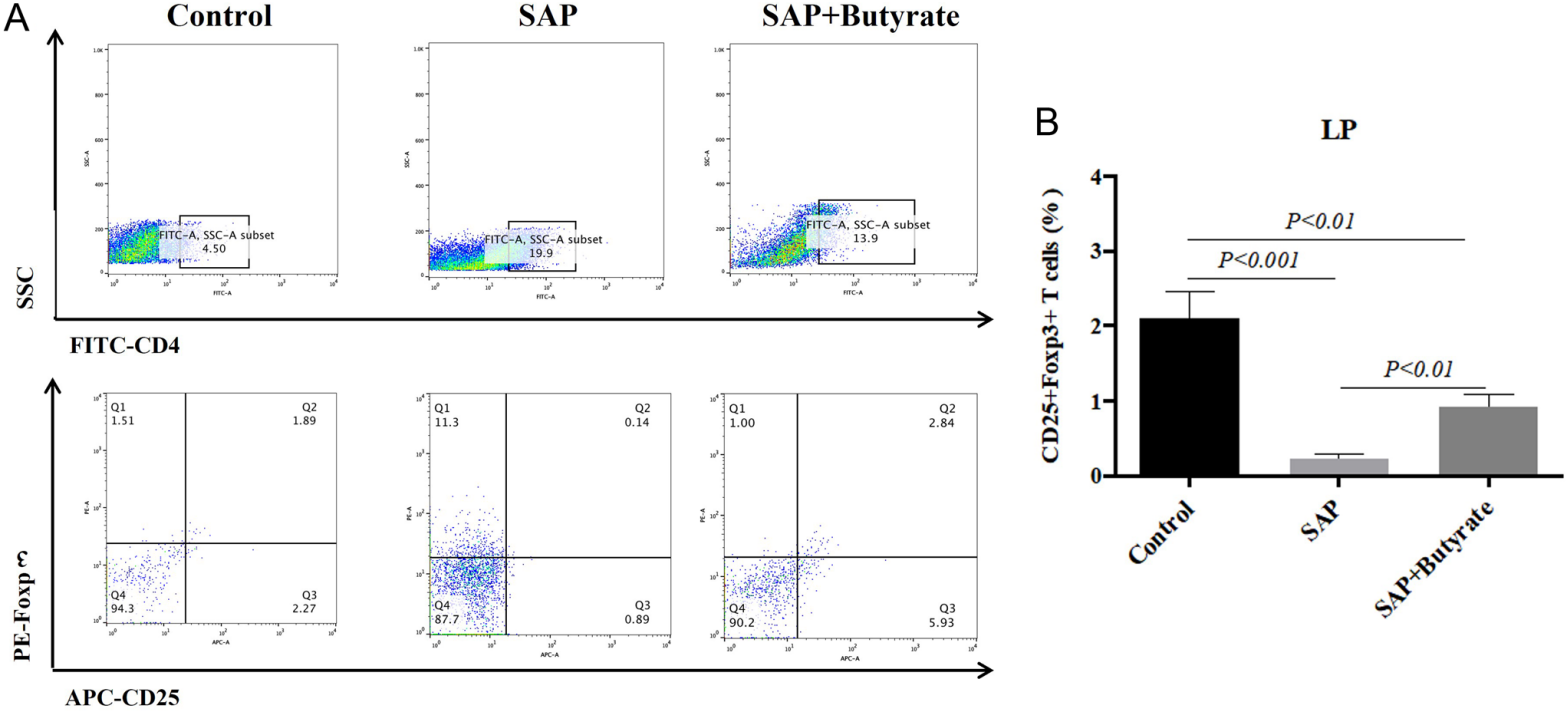
The effect of butyrate on the percentage of CD4 + CD25 + Foxp3 + Treg cells in the lamina propria. Control: Control group, SAP: SAP group, SAP + butyrate: SAP + butyrate group. (A) The percentages of CD4 + CD25 + Foxp3 + Treg cells detected by flow cytometry analysis. (B) Quantification of the percentages of CD4 + CD25 + Foxp3 + Treg cells in each group. Control group versus SAP group: *P* < .001, Control group versus SAP + butyrate group: *P* < .01, SAP group versus SAP + butyrate group: *P* < .01. n = 8 in each group. SAP, severe acute pancreatitis.

In addition, we also performed flow cytometry analysis to show the change of T lymphocyte subsets in the LP. Decreased percentage of CD4 + CD25 + Foxp3 + Treg cells was observed in the rats of the SAP group compared to those in the control (*P* < .001). However, compared to the rats in the SAP group, rats in the SAP + butyrate group showed upregulated percentage of CD4 + CD25 + Foxp3 + Treg cells (*P* < .01, Figure 6A and B), which was consistent with mitigated gut inflammation.

### Butyrate Decreased Apoptosis of Intestinal Epithelial Cells in SAP Rats

**Supplementary Figure 4.**
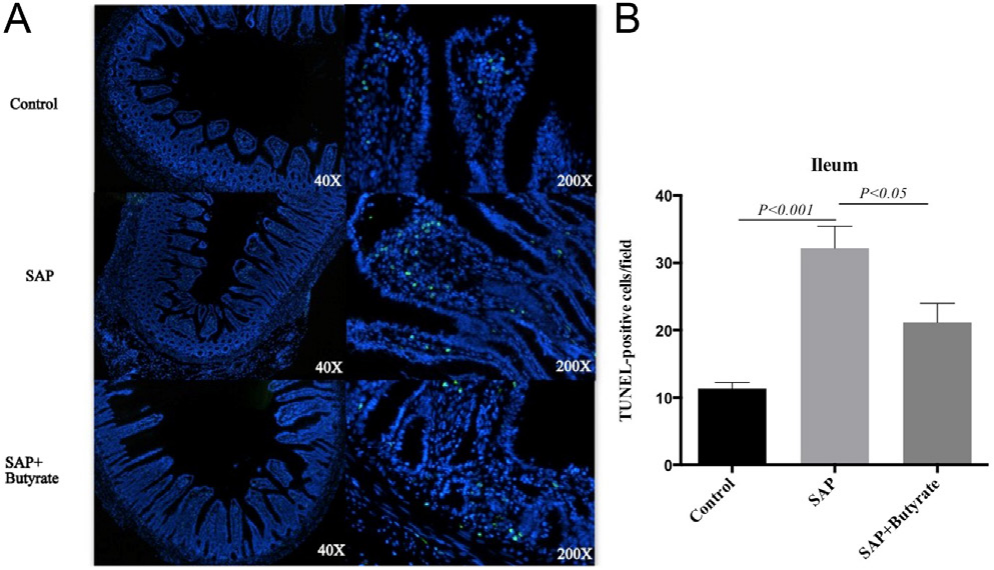
The effect of butyrate on the apoptosis of epithelial cells in the ileums. Control: Control group, SAP: SAP group, SAP + butyrate: SAP + butyrate group. (A) The Terminal-deoxynucleoitidyl Transferase Mediated Nick End Labeling (TUNEL) detection of apoptotic cells in the ileums. Green particles represented the positive change. (B) Quantification of the apoptotic cells in each group. Control group versus SAP group: *P* < .001, Control group versus SAP + butyrate group: *P* > .05, SAP group versus SAP + butyrate group: *P <* .05. n = 8 in each group. SAP, severe acute pancreatitis.

**Supplementary Figure 5.**
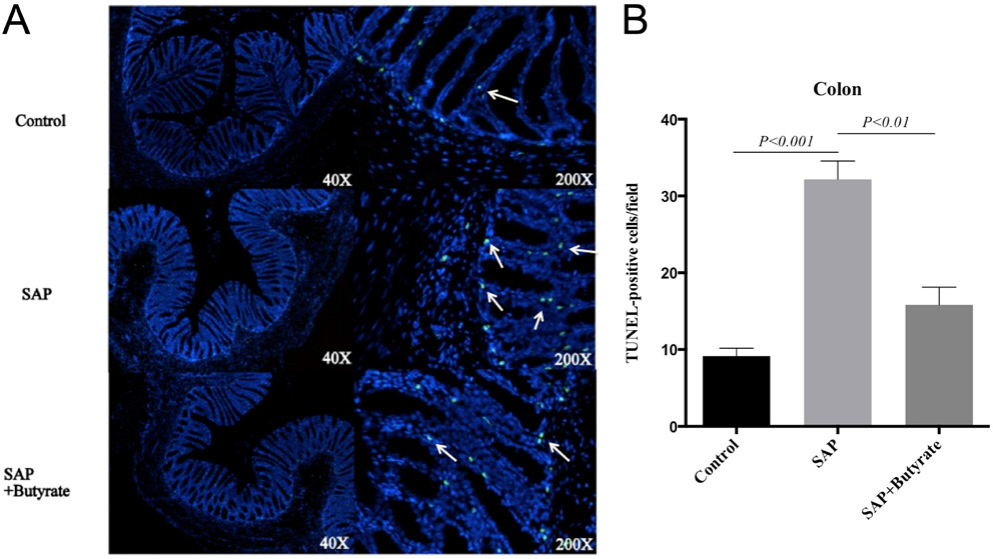
The effect of butyrate on the apoptosis of epithelial cells in the colons. Control: Control group, SAP: SAP group, SAP + butyrate: SAP + butyrate group. (A) The TUNEL detection of apoptotic cells in the colons. Green particles (arrow) represented the positive change. (B) Quantification of the apoptotic cells in each group. Control group versus SAP group: *P* < .001, Control group versus SAP + butyrate group: *P* > .05, SAP group versus SAP + butyrate group: *P <* .01. n = 8 in each group. SAP, severe acute pancreatitis.

Lastly, the apoptosis of intestinal epithelial cells was assessed by performing TUNEL assay. The increased density of apoptotic cells was observed in the intestines of the rats in the SAP group (*P* < .001 for ileum and colon, Supplementary Figures 4A-B and 5A-B), which was decreased significantly in rats of the SAP + butyrate group (*P* < .05 and *P* < .01 for ileum and colon, respectively).

## Discussion

The previous study of the authors demonstrated that gut mucosal dysfunction occurred at the initial stage of AP, and that it was correlated with disease severity and worse outcome.^25^ Besides, gut-based therapies (e.g., early enteral nutrition and intestinal decontamination) have shown their beneficial effects on AP patients.^26,27^ It is therefore suggested that early gut barrier injury might be critical to the development of the SAP-related complications (e.g., sepsis, infected pancreatic necrosis (IPN) and MODS). In addition, recent studies suggested that immune cells appeared to play a vital role in the pathogenesis of AP and could determine disease severity,^8^ which demonstrated that AP might also be an immune disorder disease, instead of an inflammatory disorder disease. This study aimed to identify the functional effect of oral butyrate to the intestinal barrier dysfunction and the local immune response in the SAP rat model. The present study demonstrated that oral butyrate could mitigate SAP-induced intestinal barrier injury, as reflected by downregulated histopathological scores and upregulated epithelial tight junction (TJ) expressions; it also exerted an immunomodulatory effect.

Butyrate, a 4-carbon SCFA, refers to one of the critical microbial fermentation products.^28^ Butyrate has been reported as one of the vital components in maintaining colon health. According to existing studies, butyrate metabolic defects contributed to the development of colon inflammatory diseases.^29,30^ Besides, butyrate exerted anti-oxidant, anti-inflammatory, and anti-carcinogenic effects in vitro and in vivo based on several mechanisms.^31-33^ The inhibition of nuclear factor kappa B (NF-κB) and histone deacetylase (HDAC) has been the most studied mechanism of butyrate exerting anti-inflammatory effect.^34,35^ However, recent studies revealed that immune modulation might be another significance mechanism of butyrate in mitigating intestinal and colonic inflammation. According to the study by Furusawa et al^36^ butyrate could mitigate the development of colitis in a mice model by inducing the differentiation of colonic Treg cells.^36^ Consistent with Furusawa’s study, this study also found that butyrate could elevate the percentage of Treg cells in both ileum and colon and then reduce the inflammatory response. A previous study confirmed that GPR109A (encoded by *Niacr1*), a receptor for butyrate in the colon, could enhance anti-inflammatory properties in colonic antigen presenting cells and induce the differentiation of Treg cells indirectly.^37^ In the present study, the variation of GPR109A in 3 groups was also found to be consistent with Foxp3.

A previous study suggested that epithelial apoptosis was promoted under colitis and Crohn’s disease, which led to the further gut barrier loss.^38^ This indicated that apoptosis might be another vital mechanism for gut barrier dysfunction in inflammatory disorder diseases. Furthermore, an in vitro study by Bailon^32^ suggested that low concentrations of butyrate would not induce the apoptosis of differentiated epithelial cells.^32^ The present study also revealed that butyrate could significantly reduce the apoptosis of the epithelial cells in ileum and colon of the SAP rats and thus prevent gut barrier loss.

Treg cells, that is, regulatory T cells, refer to a subset of CD4 + T cells highly expressing IL-2α (CD25) and Foxp3. Treg cells are capable of helping maintain the intestinal homeostasis by preventing inappropriate innate and adaptive immune responses.^39,40^ As reported from Boehm et al. depletion of Foxp3 + Treg could obviously aggravate the intestinal inflammatory responses in C57BL/6 mice, which further demonstrated the significance of Foxp3 + Treg to the immune balance of the intestine. The present study also indicated that with the progress of the inflammatory response in the intestine, the percentage of intestinal Treg cells was markedly reduced after the SAP modeling. Moreover, butyrate could elevate the percentage of Treg cells and mitigate the inflammatory responses in the intestines of SAP rats, which demonstrated that excessive immune response might be one of the causes for gut barrier injury in SAP and the protective effect of butyrate might be exerted by regulating the immune response of the intestine. However, in-depth studies should be conducted to verify the potential mechanism.

## Conclusion

In brief, butyrate could significantly mitigate the intestinal injury induced by SAP and maintain the gut barrier function, probably by inducing the differentiation of Treg cells. Subsequent research is required to verify the exact effect of Treg cell in SAP-induced intestinal injury.
